# Silk Assembly against Hydrophobic Surfaces—Modeling and Imaging of Formation of Nanofibrils

**DOI:** 10.1021/acsabm.2c00878

**Published:** 2023-02-15

**Authors:** Danilo Hirabae De Oliveira, Michal Biler, Carsten Mim, Linnea Enstedt, Mathias Kvick, Patrick Norman, Mathieu Linares, My Hedhammar

**Affiliations:** † Department of Protein Science, School of Engineering Sciences in Chemistry, Biotechnology and Health, KTH Royal Institute of Technology, AlbaNova University Center, SE-106 91 Stockholm, Sweden; ‡ Division of Theoretical Chemistry and Biology, School of Engineering Sciences in Chemistry, Biotechnology and Health, KTH Royal Institute of Technology, SE-100 44 Stockholm, Sweden; § Department of Biomedical Engineering and Health Systems, Royal Technical Institute (KTH), Hälsovägen 11C, SE-141 27 Huddinge, Sweden; ∥ Spiber Technologies AB, SE-114 28 Stockholm, Sweden; ⊥ Laboratory of Organic Electronics and Scientific Visualization Group, ITN, Linköping University, SE-581 83 Linköping, Sweden

**Keywords:** spider silk, spidroin, MaSp, hydrophobic surfaces, nanofibrils self-assembly, atomic force microscope, cryo-electron microscopy, molecular dynamics modeling

## Abstract

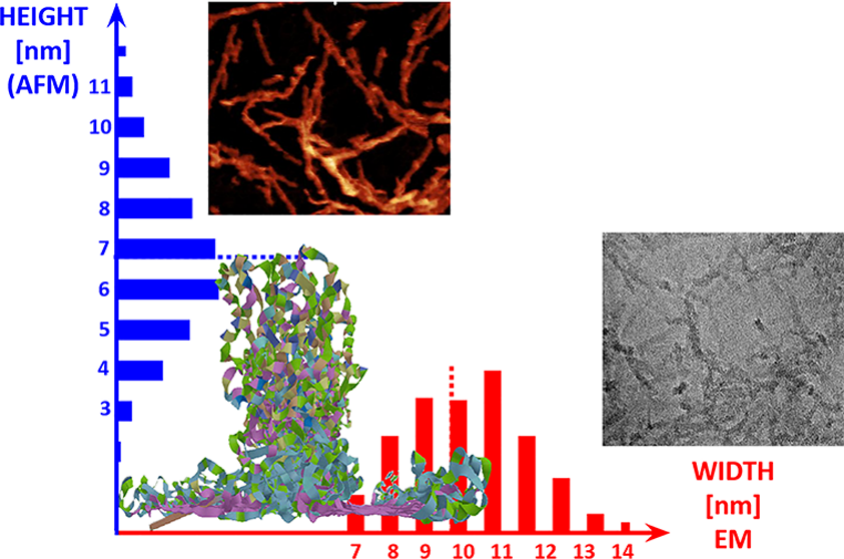

A detailed insight about the molecular organization behind spider silk assembly is valuable for the decoding of the unique properties of silk. The recombinant partial spider silk protein 4RepCT contains four poly-alanine/glycine-rich repeats followed by an amphiphilic C-terminal domain and has shown the capacity to self-assemble into fibrils on hydrophobic surfaces. We herein use molecular dynamic simulations to address the structure of 4RepCT and its different parts on hydrophobic versus hydrophilic surfaces. When 4RepCT is placed in a wing arrangement model and periodically repeated on a hydrophobic surface, β-sheet structures of the poly-alanine repeats are preserved, while the CT part is settled on top, presenting a fibril with a height of ∼7 nm and a width of ∼11 nm. Both atomic force microscopy and cryo-electron microscopy imaging support this model as a possible fibril formation on hydrophobic surfaces. These results contribute to the understanding of silk assembly and alignment mechanism onto hydrophobic surfaces.

## Introduction

Spider silk has been claimed as the utmost biopolymer surpassing the mechanical properties of many modern synthetic fibers.^1−3^ In addition to the tensile strength and elasticity, silk has become attractive due to good biocompatibility and natural degradation. Thus, various ways of developing silk-based materials are being explored for biomedical applications, such as tissue regeneration,^4,5^ wound dressings,^6^ and drug delivery.^7^

Spider silk is made of sophisticated proteins, called spidroins, that are produced and stored in glands in the back of the spider. Spidroins are large proteins (size ranging from 200 to 350 kDa) composed of three main segments: a central repetitive region flanked by non-repetitive and conserved N- and C-terminal domains. Spidroins have a unique property to rearrange themselves into silk fibers. The repetitive region is the largest part, with the order of hundred repetitions of poly-alanine stretches and glycine-rich repeats, which constitute the major part of the silk fiber composition.^8,9^ The specialty and mechanical attributes among the different silk types diverge according to variations within these repetitive blocks. The N- and C-terminal domains (NT and CT) are 100 to 150 residues long and phylogenetically preserved.^10,11^ Among the diverse categories of silk proteins, the major ampullate spidroin 1 (MaSp1) makes up the main component of the dragline silk.

During the spinning process, the spidroins experience a secondary structure transition, in which stretches of alanine in the repetitive region assemble into anti-parallel β-sheets oriented along the fiber axis.^12,13^ The alignment and stacking of such motifs resolve into β-nanocrystalline structures and contribute to the stiffness of silk.^14,15^ The fiber β-sheet crystalline component has been previously described by X-ray nanodiffraction,^16^ Raman spectroscopy,^17^ Fourier transform infrared spectroscopy (FTIR),^18^ and nuclear magnetic resonance.^19^ Furthermore, mesoscopic analysis of silk has revealed that these β-nanocrystalline structures are embedded in an amorphous polypeptide network mainly composed of glycine-rich sequences, which contributes to silk’s elasticity.^20,21^

Recombinant production of selected parts of silk proteins in *E. coli* has contributed to a partial understanding of the hierarchical structures of silk. Motifs, such as GPGXX and GGX, from the repetitive region have been shown to form β-turns and 3_10_-helical secondary structures, respectively.^22−24^ NT has been shown to attain a five-helix bundle subunit that forms an antiparallel homodimer as the solution becomes slightly acidic.^25^ CT has also been shown to adopt a five-helix fold in solution but intertwisted into a parallel homodimer linked by an interchain disulfide bond and stabilized by intramolecular salt bridges.^26^

A partial spidroin, 4RepCT, consisting of four repetitions of stretches of alanines (12–15) and glycine-rich segments followed by the CT derived from *Euprosthenops australis* dragline silk, has the ability to self-assemble into macroscopic silk-like fibers at the air–water interface under physiological conditions.^27,28^ Inclusion of the NT is not necessary for silk formation but has been shown to allow control of the speed of fiber formation using a pH shift.^25,29^ The recombinant spider silk protein 4RepCT has thus emerged as a distinct simple silk model, which in fiber form has been evaluated by diverse biophysical methods like circular dichroism, FTIR, and fiber X-ray diffraction.^27,28,30^ Recent studies suggest that the 4RepCT spider silk protein (as well as the repetitive part alone) adsorbs on hydrophobic surfaces, triggering a large-scale protein conformational change that results in the formation of nanofibrils.^31,32^ Promoted by hydrophobic interactions, the silk protein partly attains β-sheet structures. Such a condition is critical to fibril nucleation initiation. The fact that silk nanofibrils can be formed by self-assembly on hydrophobic surfaces opens up for a facile method for the formation of stable silk coatings that could be useful in various industrial applications.

The potential for silk proteins to self-assemble into nanofibrils at various surfaces and interfaces has previously been investigated experimentally, contributing to an initial understanding of the prerequisites and mechanisms behind the related structural rearrangements of the proteins.^31,32^ The next step in order to understand the mechanism better is to simulate the structural rearrangements to find the most likely molecular refolding and arrangement. This understanding will be important in order to further explore the usability of silk coatings by, for example, suggesting what parts of the proteins that are exposed to the surroundings and mostly suitable for functionalization.

Fine-scale modeling of other silk structures has been done mainly through all-atom and coarse-grained molecular dynamics (MD) simulations.^33^ As most MD simulations are limited to timescales of a few hundred nanoseconds, typically selected parts of the repetitive region have been used instead of the very long full-length spidroins. For example, MD has given insights into how the self-assembly network is affected by shear forces and variations of the repetitive blocks.^34,35^ To our knowledge, there are no previous reports about MD simulations of the CT. Moreover, previous studies have modeled the silk sequences without any alignment in space. The adsorption of silk to hydrophobic surfaces and thereby induced structural change correlation remains unclear.

In this study, we use Amber Tools Molecular Dynamics Package to process atomistic models that illustrate the conversion from soluble proteins to macromolecular arrangement of silk nanofibrils on surfaces. As silk sequence models, we use the partial spidroins 4Rep, CT, and 4RepCT, and 1-undecanethiol and 11-mercapto-1-undecanol are used to represent hydrophobic and hydrophilic surfaces, respectively. The results describe a large-scale periodic system proposed for the formation of nanofibrils of 4RepCT on a hydrophobic surface. The modeling results are complemented with the assessment of the size of 4RepCT nanofibrils formed on hydrophobic surfaces, as analyzed from images obtained experimentally using atomic force microscopy (AFM) and electron microscopy (EM).

## Materials and Methods

### Construction of Surfaces

**Figure 1:**
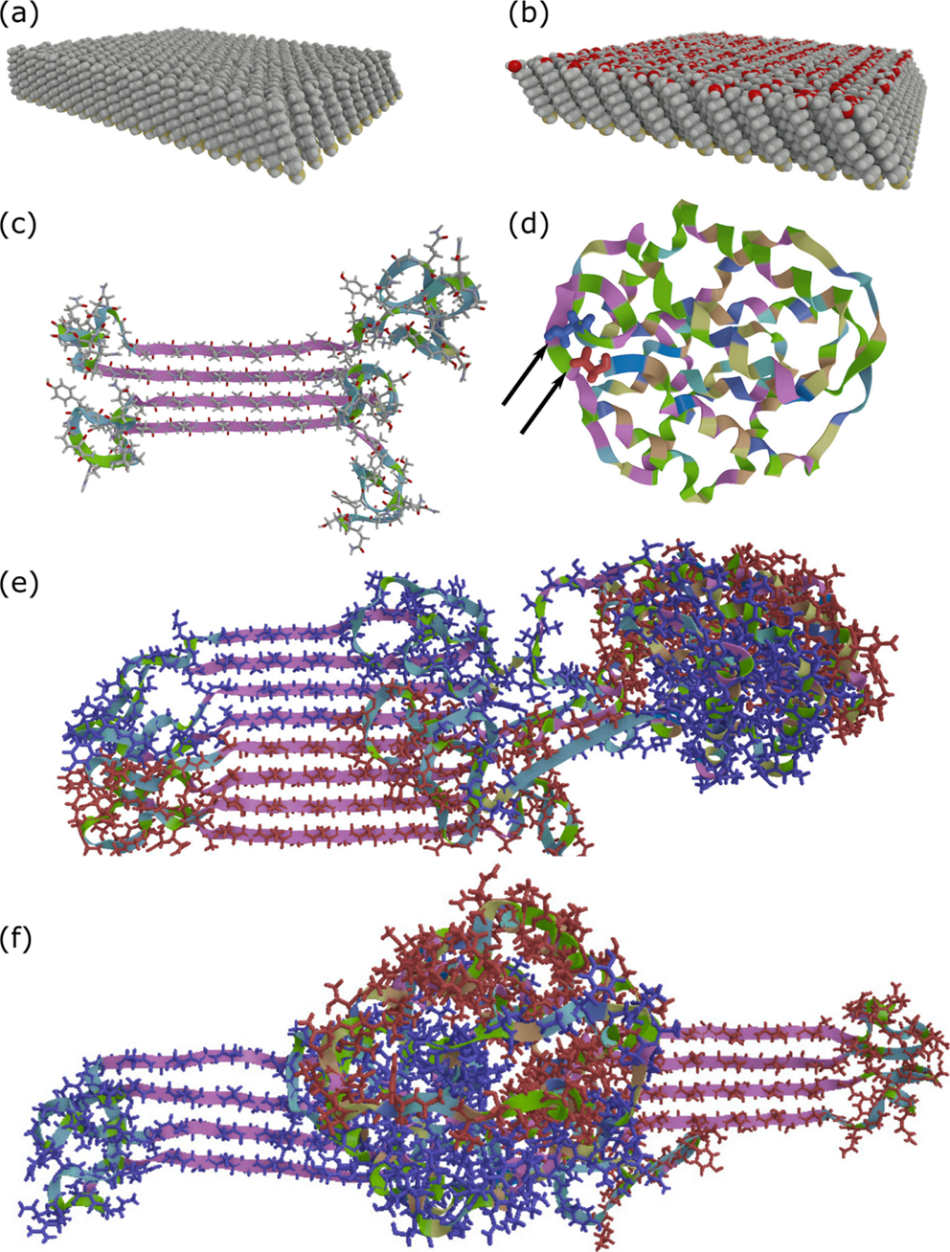
Illustration of surfaces and silk protein building blocks used within the study. (a) Hydrophobic 1-undecanethiol and (b) hydrophilic 11-mercapto-1-undecanol surfaces with an inter-sulfur distance of 4.97 Å and alkane tilt angles of *ca.* 30°. (c) 4Rep with β-sheets of alanines (purple) glycine-rich random-coil loops (green). (d) Dimer of CT with the two N-terminal residues (where 4Rep is linked) marked in blue and red. (e) Alongside arrangements and (f) wing arrangement of a dimer of 4RepCT.

Hydrophobic surfaces were constructed of 1-undecanethiol, while hydrophilic surfaces were made of 11-mercapto-1-undecanol. Both molecules have a SH group on the carbon linker side, and CH_3_ (hydrophobic) or OH (hydrophilic) group on the other side (Figure 1a,b). The SH groups were fixed in a hexagonal arrangement^36^ in order to mimic attachment to a solid surface. The surfaces were built in sizes of 10 × 10, 15 × 15, and 25 × 25 nm.

### Construction of Silk Protein Building Blocks

In order to build the 4Rep part of the 4RepCT silk protein (for sequence see Figure S1), we first built four chains of alanines (12–15) in β-sheet alignment by building four separate chains of 12, 15, 15, and 14 alanine residues, respectively, in tleap using the Amber Molecular Dynamics Package, and moved them in VMD^37^ in a way to form an antiparallel β-sheet arrangement. To create the rest of 4REP (glycine-rich loops between alanine stretches), we used PEP-FOLD3,^38−40^ which predicts small peptide structures according to amino acid sequence. Avogadro^41^ was used to join the loops to the β-sheet core. The minimized final structure of 4Rep is shown in Figure 1c.

To build the CT part of the 4RepCT silk protein, we used the crystallographic structure of the C-terminal domain of another spider silk protein, ADF-3 (PDB ID: 2KHM) from the protein databank (www.rcsb.org), and modified the amino-acid sequence according to our version (Figure 1d). The 51 amino acids that differed in 2KHM were exchanged by the use of the protein structure homology modeling server “SWISS-MODEL”.^42−44^ CT is a homo dimer joint together by disulfide-bridge between cysteine residues, which was explicitly written (by bond command in tleap) when creating topology files with LEaP implemented in Amber.

4Rep and CT parts were then joined together by syncing the sequences (4 residues overlap).

Because for one dimeric CT, there are two 4Rep parts, we built 4RepCT in two different arrangements: (i) alongside the arrangement, where two 4Rep parts are alongside, and the CT part is next to them and (ii) wing arrangement, where two 4Rep parts are opposite to each other, and the CT part is in the middle (Figure 1e,f).

### MD Simulations

Geometry optimization of the main building blocks of both hydrophobic and hydrophilic surfaces, followed by RESP^45^ charge calculations were carried out using the Kohn–Sham density functional theory in conjunction with the B3LYP exchange–correlation functional^46^ and the cc-pVDZ basis set and using the Gaussian09 program.^47^ Then, topologies and initial coordinates were created using the LEaP program with the general amber force field^48^ (GAFF) and the ff14SB force field^49^ for the surface and protein molecules, respectively. Explicit solvent molecules were described using the TIP3P water model.^50^ MD simulations were performed for defined time frames (50–250 ns) at defined temperatures (300 or 450 K) using the Amber Molecular Dynamics Package.^51,52^ The systems were first minimized and relaxed. Then, the MD simulations were performed using Langevin dynamics and constant pressure periodic boundary with an average pressure of 1 atm and with isotropic position scaling using a Berendsen barostat.^53^ The simulations were run with a time step of 2 fs, the trajectories were recorded every 5000 steps, and the cut-off were set to 12 Å for non-bonded interactions. Snapshots from MD simulations used in the figure have been produced with VIA-MD software.^54^

### AFM Analysis

AFM measurements were performed in order to quantify the height of silk nanofibrils formed from 4RepCT silk proteins assembled on a hydrophobic surface. The 4RepCT protein solution was diluted with 20 mM Tris buffer to 0.1 mg/mL and 1 mL was placed into a well in a 24 well-plate. A hydrophobic glass coverslip [custom-made siliconized hydrophobic coverslips (Paul Marienfeld GmbH & Co. KG)] was rinsed with ethanol followed by Milli-Q water and placed in the well. After 60 min of incubation, the coverslip was removed and washed with 3 × 1 mL 20 mM Tris before it was transferred to a new well and kept in Tris buffer until the measurement. The AFM measurements were performed the day after the samples were prepared. AFM imaging was done with a Bruker Dimension FastScan instrument, using ScanAsyst Fluid+ tips (nominal tip radius 2 nm) and PeakForce tapping. The measurements were performed under wet conditions, that is, the samples were never dried. The fibrils were detected using a steerable filter; at each location, where a fibril was detected, a small part of the image was cut out and rotated to a set direction. The mean fibril height was then constructed from by adding up the extracted parts.

### EM Analysis

EM measurements were performed in order to quantify the width of the silk nanofibrils formed from 4RepCT silk proteins assembled on a hydrophobic surface.

The 4RepCT protein solution was diluted with 20 mM Tris buffer to 0.1 mg/mL in a 96 well-plate. Holey carbon grids precoated with graphene oxide (EM Resolutions, cat no GOHC300Cu25) were glow charged (5 s, 20 mA, Easiglow; Ted Pella) to improve buffer retention. To adsorb the silk nanofibrils, grids were placed on the top interface of the silk protein solution. After 30 s, the silk protein solution was wicked away using a filter paper. The sample was washed by bringing the surface in contact with a droplet of Tris buffer, followed by 2 droplets of Milli-Q, and lastly a longer contact of 15 s with Milli-Q. After each wash excess liquid was wicked away using a filter paper. After the last wash, the sample was vitrified with a Vitrobot Mark 1 (Thermo Fisher) and the sample was imaged on a JEOL 2100f (JEOL) at 200 kV and with a TVIP camera (Tietz) and a pixel size of 1.36 Å, or a DE20 camera (DirectElectron) with a pixel size of 1.04 Å. The movies recorded with the DE20 camera received a total dose of 120 electrons per Å^2^ fractioned over 32 frames. The movies were motion corrected and dose weighted with MotionCorr.^55^ The micrographs from the TVIPS camera were evaluated with ImageJ.^56^ The thickness of individual fibrils (*n* = 330) from 15 micrographs were manually measured. For visualization, the width was organized in a histogram. For 2D classification of fibres (from the DE20 dataset), we used the Scipion suite^57^ and processed the data with relion 2.^58^ In short, unbranched fibrils were segmented (208 Å box size with 90% overlap), resulting in 985 segments. These segments were classified in 2D. The major class represents ∼70% of all segments.

## Results and Discussion

### MD Simulations Show How the Different Silk Protein Parts Are Affected by Different Surfaces

**Figure 2:**
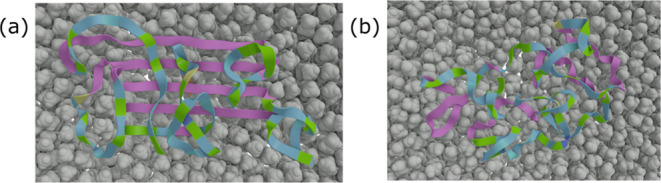
MD simulations of 4Rep in β-sheet conformation. Structure of 4Rep after 100 ns simulations at 450 K on (a) hydrophobic and (b) hydrophilic surfaces showing structure integrity in the former case and disintegration in the latter case.

MD simulations of the 4Rep part with alanine stretches in β-sheet conformation were performed on both hydrophobic and hydrophilic surfaces. At lower temperatures (300 K), no structural changes were induced on any of the surfaces, during 125 ns (Table S1). To accelerate structural changes while not adding an unrealistic amount of kinetic energy, the temperature was increased to 450 K. On the hydrophobic surface, the alanine blocks of 4Rep remained in a β-sheet arrangement along the surface, while the loops move up on top of the alanine sheets (Figure 2a). However, on the hydrophilic surface, the β-sheet structure was destabilized in less than 10 ns and underwent structural changes (Figure 2b). When two 4Rep parts were placed next to each other, a similar behavior of stabilization of β-sheets on the hydrophobic surface and destabilization on the hydrophilic surface was observed. This is in line with previous experimental findings that 4Rep/4RepCT adhere and assemble into β-sheet aggregates/fibrils on hydrophobic but not hydrophilic surfaces (Figure S2, refs (31) and (32)).

For similar MD simulations of CT, the protein domain was slightly stabilized by H-bonding and electrostatic interactions on the hydrophilic surface but not affected by the hydrophobic surface (Table S1). Nevertheless, no specific behavior in terms of a secondary structure was seen, and the CT kept the alpha-helical structure along the whole MD simulation (200 ns at 300 K) on both hydrophobic and hydrophilic surfaces (Figure S3).

### Dimeric Form of 4RepCT Is Modeled in Two Different Arrangements

From experimental studies, 4RepCT is known to form a dimer in solution.^12^ For building models of 4RepCT, which should, thus, contain one dimeric CT and two 4Rep parts, we used two different arrangements: (i) alongside arrangement, where two 4Rep parts are alongside, and CT part is next to them and (ii) wing arrangement, where two 4Rep parts are opposite to each other, and CT part is in the middle (Figures 1e,f and S4).

**Figure 3:**
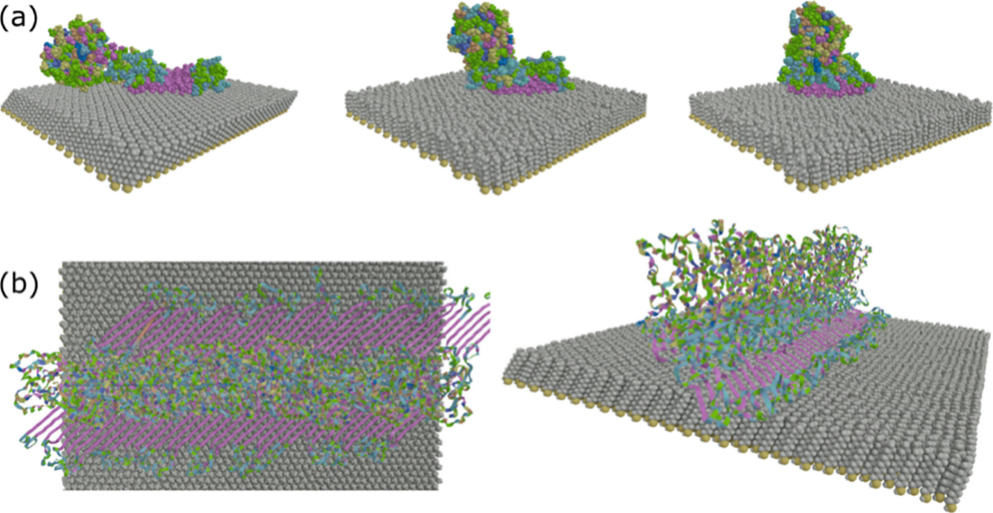
Evolution of 4RepCT on a hydrophobic surface. (a) Time-progression during 50 ns at 450 K showing CT motion to a position on-top of 4Rep for the alongside arrangement. Height of ∼5.1 nm and a width of ∼4.3 nm in the last frame. (b) Final stable structure of a periodic fibril made of nine 4RepCT dimers in a wing arrangement, showing a height of ∼7 nm and a width of ∼11 nm.

When modeling 4RepCT in an alongside arrangement on the hydrophobic surface, the CT domain goes on top of the 4Rep parts and is stabilized by H-bonding interactions (Figure 3a). Moreover, the loops in the 4Rep part form a saddle-shaped structure allowing CT to go on top of this. It is very clear that the CT domain is averse to being on the hydrophobic surface. On the hydrophilic surface, the β-sheets of 4Rep are destabilized while CT still keeps its alpha-helical structure and does not interact with the hydrophilic surface (Figure S5). Simulation were run at 450 K for 50 and 40 ns on hydrophilic and hydrophobic surfaces, respectively.

Because the CT domain prefers to interact with the 4Rep part compared to the surfaces, the 4RepCT in an alongside arrangement would yield structures that are narrower than what would be expected from previous AFM images.^13^ In this arrangement, it is also difficult to see how the periodicity needed for fibril formation would be stabilized. Therefore, we decided to further investigate the option of dimeric 4RepCT in a wing arrangement. Because the hydrophilic surface is obviously not in favor of keeping β-sheet structures of the alanine residues in the 4Rep part, we decided to perform a MD simulation of 4RepCT in the wing arrangement only on the hydrophobic surface. Interestingly, when the MD simulation starts from the wing arrangement (two 4Rep parts being opposite to each other with CT being in the middle), the CT part varies between being slightly up and down closer to the 4Rep part (Figure S6). The 4Rep parts thereby get into a parallel configuration within the simulation time (25 ns at 450 K). To build a periodic system needed for representing the fibril form, nine repetitions of 4RepCT dimers in the wing arrangement were placed on a hydrophobic surface (Figure 3b). The configuration was found to be stable over the whole 15 ns of simulation at 300 K.

### AFM and EM Show Fibril Sizes Corresponding to 4RepCT in Wing Arrangement

**Figure 4:**
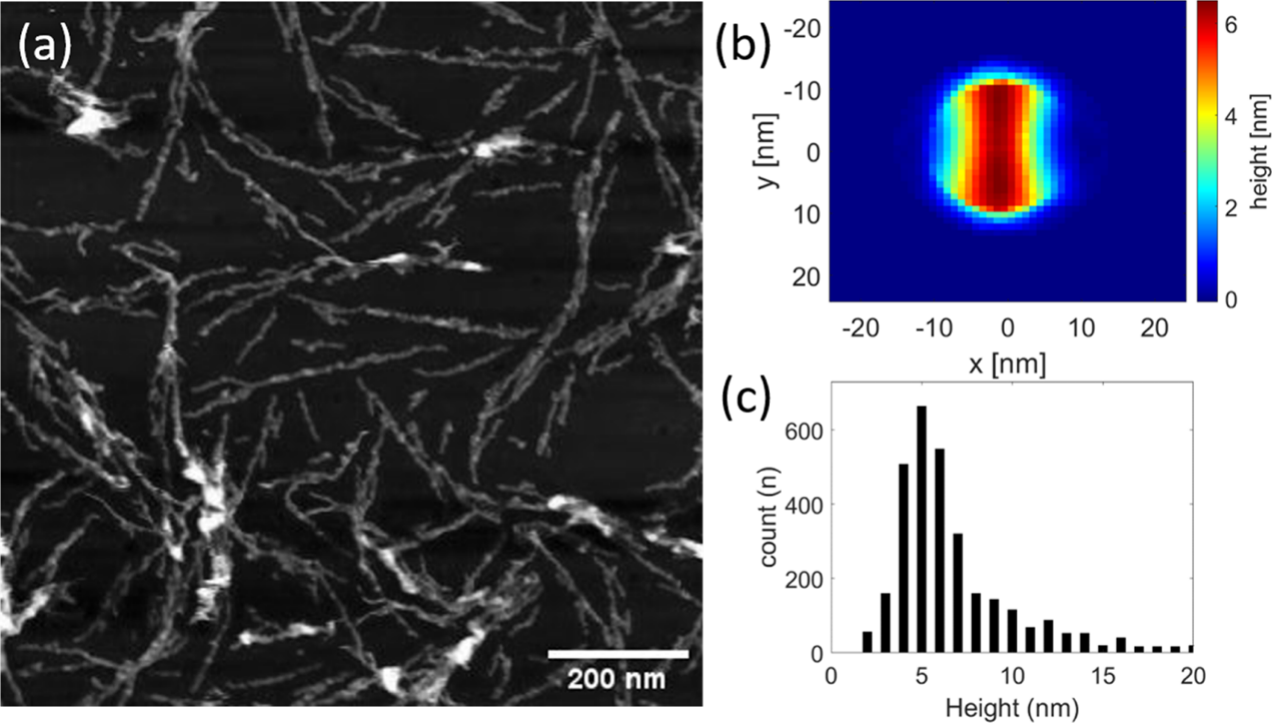
Atomic force micrograph of nanofibrils self-assembled from 4RepCT on a hydrophobic surface. (a) Original AFM image. Scale bar 200 nm. (b) Mean fibril extracted from the image. (c) Histogram of the measured height of individual fibril segments (*n* = 3132).

AFM and cryo-EM measurements were performed in order to discover the size of fibrils self-assembled from solutions of 4RepCT. Hydrophobic surfaces were chosen because MD simulations did show the stabilization of β-sheets on the hydrophobic surface and destabilization on a hydrophilic counterpart, which is in line with previous experimental observations that the silk assembly is promoted on hydrophobic surfaces^31^ (Figure S2). Custom-made siliconized hydrophobic coverslips were used in order to get a smooth enough surface to get reliable measures of the silk fibrils. For such delicate structures, it is with AFM that it is difficult to assess whether the width of the structures in the images are affected by the diameter of the cantilever tip (i.e., limited image resolution). Thus, using AFM, a quantitative assessment of fibril sizes is more reliable when studying height differences than widths. This is why AFM was herein used to estimate the height of the 4RepCT nanofibrils, and cryo-EM was used to estimate the width of the nanofibrils. The AFM measurements were performed under wet conditions, on samples prepared the day before and stored in buffer, that is, the samples were never dried. Thus, AFM measurements in liquid drops allow imaging of the fibrils in their native conformation after self-assembly onto the hydrophobic surfaces. The fibrils are detected using a steerable filter, and at each location, where a fibril was detected, a small part of the image was cut out and rotated to a set direction. The mean fibril size was then constructed by adding up the extracted parts. From this, it can be concluded that the median height of the fibrils is ∼6.77 ± 2.62 nm (Figures 4, S7). This is close to the height (7 nm) of the fibrils obtained from the evolution of 4RepCT in the wing arrangement on the hydrophobic surface (Figure 3b).

**Figure 5:**
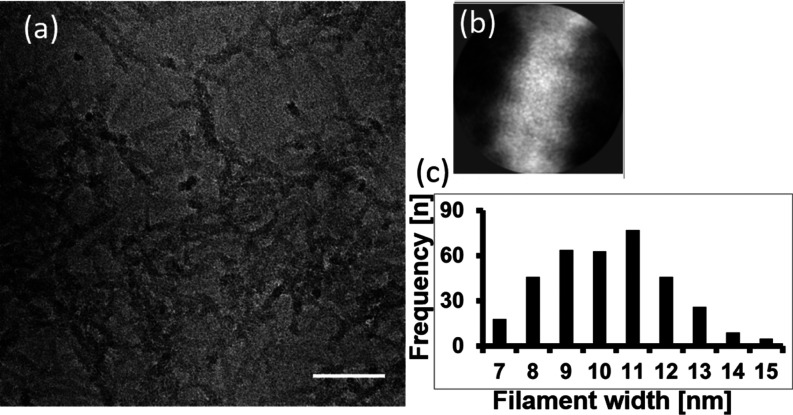
Cryo electron micrographs of nanofibrils self-assembled from 4RepCT on a hydrophobic grid surface. (a) Raw image of 4RepCT adsorbed to graphene oxide. Scale bar 100 nm. (b) Most populated 2D class average after segmentation of nanofibrils imaged by cryoEM. The box size is 20.8 nm and the fibril is about 11 nm. (c) Histogram of the measured width of individual fibrils (*n* = 330).

EM was conducted to analyze the width of the 4RepCT nanofibrils. To image the silk fibrils by EM under similar conditions as in the AFM imaging (i.e., buffer without drying), we used cryoEM. We decided to use graphene oxide-covered grids, so the fibrils can settle on a surface. The observed silk fibrils (Figure 5a) differ from amyloid fibrils (e.g., from amyloid β): no crossover was observable and the silk fibrils lack the long persistence length seen in prototypical amyloid fibrils. Although the individual fibrils are not completely straight, we attempted a 2D classification. For that, we divided the fibers into overlapping segments. We did not find that many single fibrils, which limited the number of segments for classification to 985. The 2D classes do not show any higher resolution features, yet ∼70% of the segments settle into a class with a dimeter of ∼11 nm (Figure 5b, inset). These featureless 2D classes indicate flexibility as suggested by the MD simulations. To gain better insights into the width distribution of the fibrils, we manually measured individual fibrils (*n* = 330) in 15 micrographs (Figure 5c), similar to the evaluation of the AFM data (Figure 4). The mean width of the fibrils is 9.8 ± 1.9 nm (Figure 5c), which is in good agreement with the width (11 nm) of fibrils obtained from the evolution of 4RepCT in the wing arrangement on the hydrophobic surface (Figure 3b) and the width of the most populated 2D class.

## Conclusions

**Figure 6:**
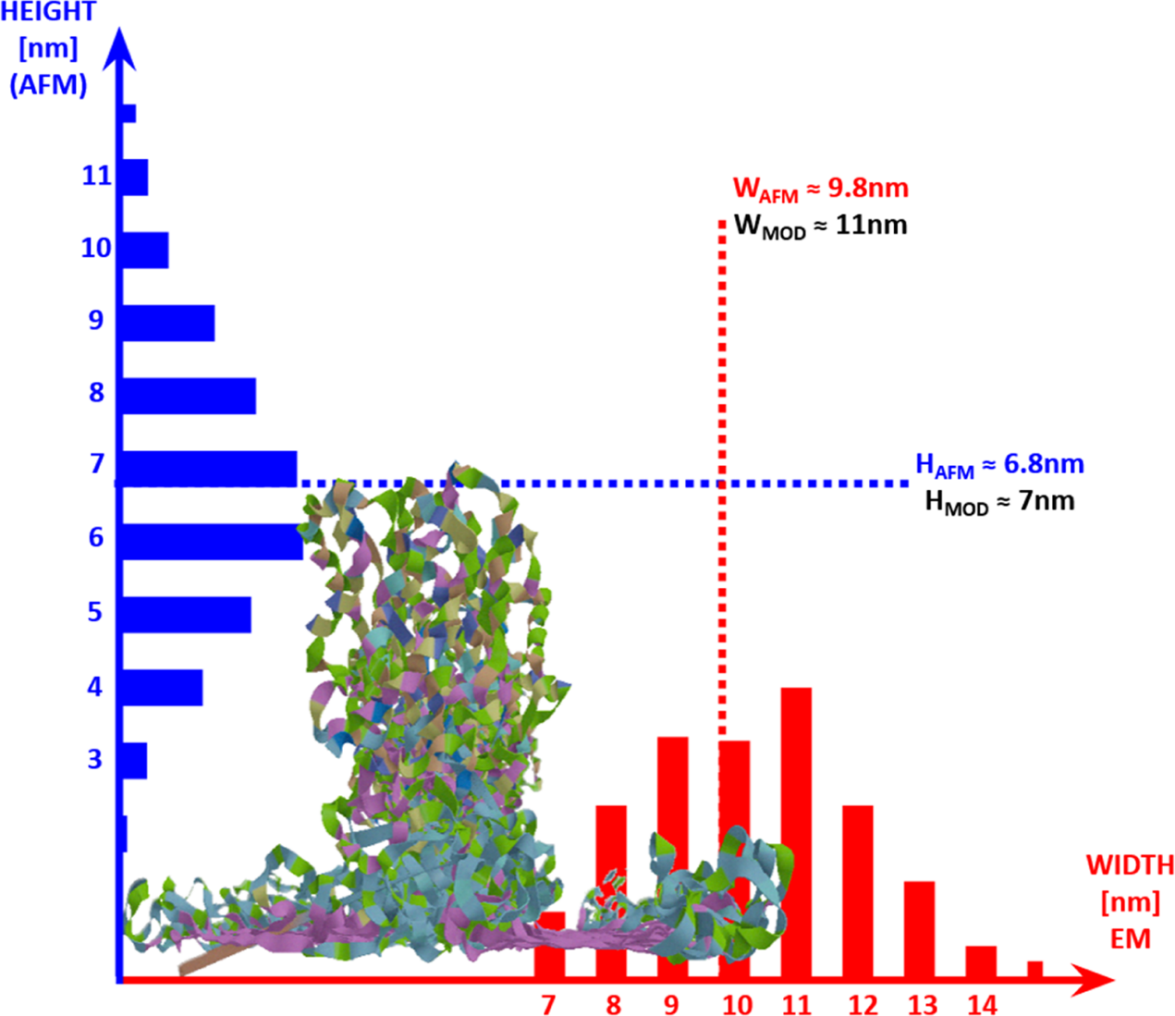
Overview of fibril height and width obtained by AFM and EM, respectively, in comparison with the wing arrangement of 4RepCT model.

Here, we use MD simulations to suggest a possible assembly route of the recombinant partial spider silk protein 4RepCT into fibrils on hydrophobic surfaces. Poly-alanine segments of the 4Rep part established β-sheet structures onto the hydrophobic surface, whereas the CT domain settled on top of the repetitive part, thereby forming a periodic oriented macrostructure. The so-called wing arrangement of the 4RepCT model attained a stable conformation on the hydrophobic surface, being in good concordance with the height and width distribution of the fibrils when compared to AFM and Cryo-EM imaging, respectively (Figure 6). This study illustrates a possible organization of different parts of spidroins, which allowed us to obtain detailed insights into silk fibril formation and structure on hydrophobic surfaces.

## Supporting Information Available

Disclose of 4RepCT protein sequence; complementary AFM images of 4RepCT on hydrophilic and hydrophobic surfaces; complementary details of CT MD simulation; presentation of suggested models for 4RepCT; evolution of 4RepCT on the hydrophilic surface and hydrophobic surface; complementary height profiles of selected fibrils of 4RepCT on a hydrophobic surface; detailed electrostatic (EELEC), and van der Waals (EVDW) energies of CT and 4RepCT during the last 10 ns of MD simulation (PDF)
