# Supplementary material for: Silk Assembly against Hydrophobic Surfaces—Modeling and Imaging of Formation of Nanofibrils
Source: ACS Appl Bio Mater. 2023 Feb 15;6(3):1011–8. doi: 10.1021/acsabm.2c00878 (PMC10031558; doi:10.1021/acsabm.2c00878)
Supplement: Supplementary file 1 — mt2c00878_si_001.pdf [file mt2c00878_si_001.pdf]

Supporting Information

Silk assembly against hydrophobic surfaces

– modelling and imaging of formation of nanofibrils

Danilo Hirabae De Oliveira, Michal Biler, Carsten Mim, Linnea Enstedt, Mathias Kvick, Patrick Norman, Mathieu Linares, My Hedhammar*

AUTHOR INFORMATION

Corresponding Author
My Hedhammar – *Department of Protein Science, School of Engineering Sciences in Chemistry, Biotechnology and Health, KTH Royal Institute of Technology, AlbaNova University Center, SE-106 91 Stockholm, Sweden.* Email: myh@kth.se

Authors
Danilo Hirabae De Oliveira – *Department of Protein Science, School of Engineering Sciences in Chemistry, Biotechnology and Health, KTH Royal Institute of Technology, AlbaNova University Center, SE-106 91 Stockholm, Sweden.*

Michal Biler – *Division of Theoretical Chemistry and Biology, School of Engineering Sciences in Chemistry, Biotechnology and Health, KTH Royal Institute of Technology, SE-100 44 Stockholm, Sweden. Current address: ICON plc. (formerly PRA Health Sciences), Kolonivägen 1, SE-226 60 Lund, Sweden.*

Carsten Mim – *Department of Biomedical Engineering and Health Systems, Royal Technical Institute (KTH), Hälsovägen 11C, SE-141 27 Huddinge, Sweden.*

Linnea Enstedt – *Department of Protein Science, School of Engineering Sciences in Chemistry, Biotechnology and Health, KTH Royal Institute of Technology, AlbaNova University Center, SE-106 91 Stockholm, Sweden.*

Mathias Kvick – *Spiber Technologies AB, SE-114 28 Stockholm, Sweden.*

Patrick Norman – *Division of Theoretical Chemistry and Biology, School of Engineering Sciences in Chemistry, Biotechnology and Health, KTH Royal Institute of Technology, SE-100 44 Stockholm, Sweden.*

Mathieu Linares – *Laboratory of Organic Electronics and Scientific Visualization Group, ITN, Linköping University, SE-581 83 Linköping, Sweden.*

GPNSGQGGYGGLGQGGYGQGAGSSAAAAAAAAAAAAGQGGQGQGGYGQGSGGSAAAAAAAAAAAAAAAGR GQGGYGQGSGGNAAAAAAAAAAAAAAAGQGGQGGYGRQSQGAGSAAAAAAAAAAAAAAGSGQGGYGGQGQ GGYGQSSASASAAASAA*STVANSVSRLSSPSAVSRVSSAVSSLVSNGQVNMAALPNIISNISSSVSASAP GASGCEVIVQALLEVITALVQIVSSSSVGYINPSAVNQITNVVANAMAQVMG*

Figure S1. Sequence of 4RepCT. **Bold**= 4Rep. *Cursive= C-terminal domain*

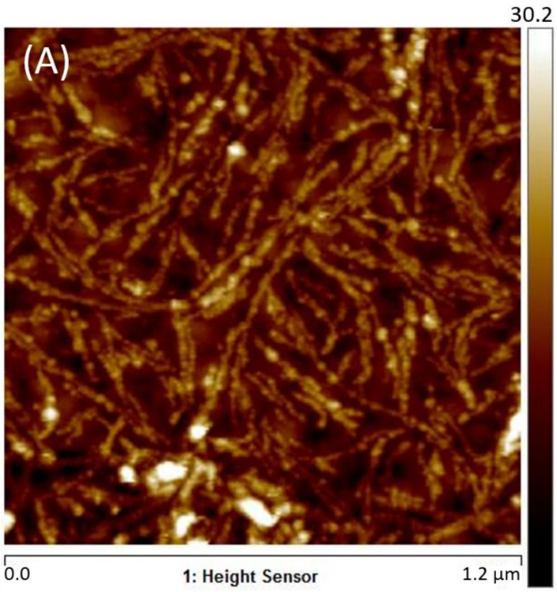

Figure S2. **Height images from Atomic force microscopy analyses of 4RepCT adsorbed onto alkylthiol-modified gold surfaces.** (a) 4RepCT adsorbed as fibrils onto a self-assembled monolayer of 1-undecanethiol, a hydrophobic surface with contact angle 100°. (b) 4RepCT did not adsorb as fibrils onto a monolayer of self-assembled 11-mercapto-1-undecanol, a hydrophilic surface with contact angle 20°. The base surfaces are made of quartz and have a sputtered gold-coating, which can be seen in both images, underneath the silk structures for the hydrophobic surface. For methods on surface preparation and sample preparation, see [31].

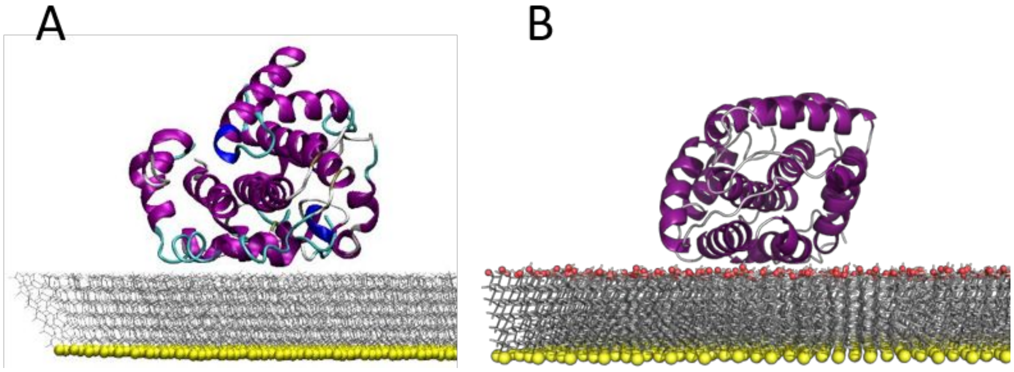

Figure S3. (A) CT on hydrophobic surface after *ca* 200 ns of MD simulation. Alpha-helices in purple, sulfur atom of the surface in yellow. (B) CT on hydrophilic surface after *ca* 200 ns of MD simulation. Alpha-helices in purple, sulfur atom of the surface in yellow and oxygen atom in red.

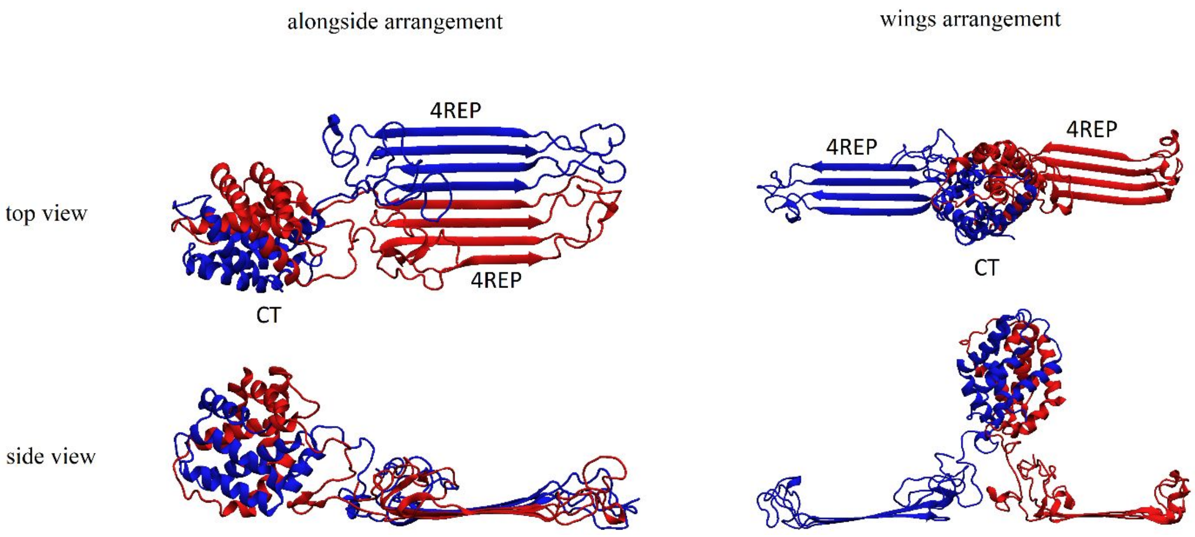

Figure S4. The two arrangements of 4RepCT, alongside (top) and wing arrangement (bottom).

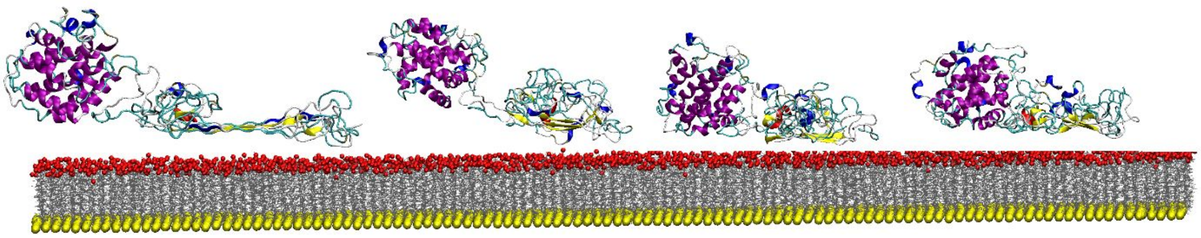

Figure S5. Evolution of alongside arrangement of 4RepCT during 40 ns at 450 K on hydrophilic surface.

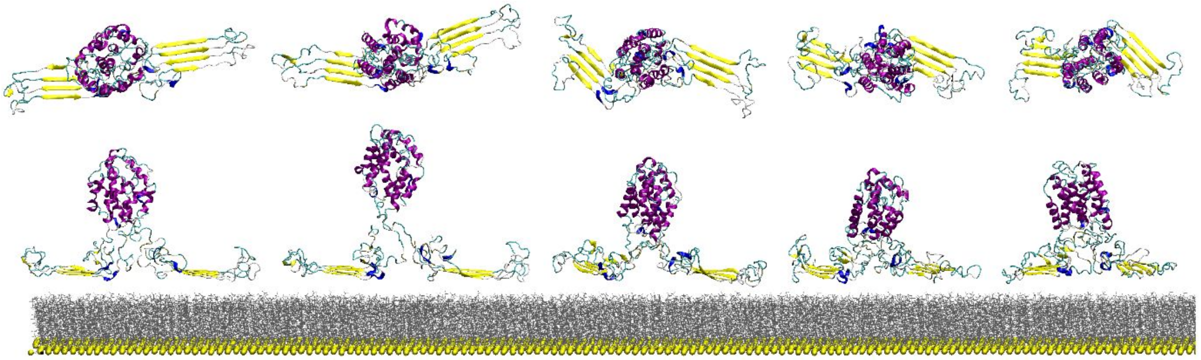

Figure S6. Evolution of 4RepCT in wing arrangement in time. Top view (top row), side view (upper row) during 25 ns at 450 K on hydrophobic surface.

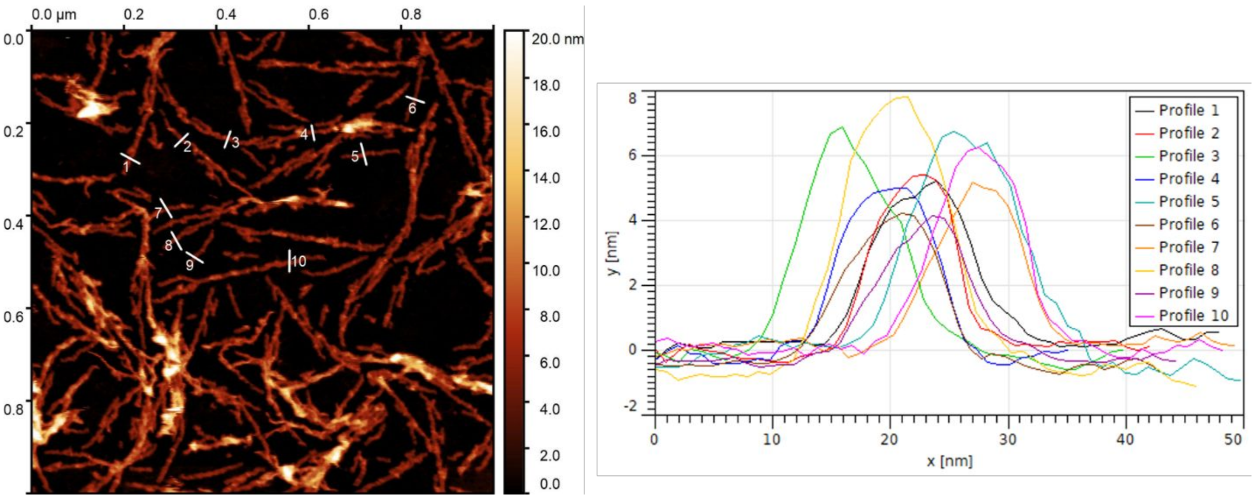

Figure S7. Atomic force micrograph (left) of nanofibrils self-assembled from 4RepCT on a hydrophobic surface, with corresponding height profiles of 10 selected fibrils (right).

	Surface	Number of H-bonds		EELEC	EVDW
		within CT	CT - surface	CT - surface	
**CT**	Hydrophilic	113	14	-149 ± 12	-133 ± 6
	Hydrophobic	117	0	-0.7 ± 0.6	-105 ± 5
**4Rep**	Hydrophilic	37	29	-355 ± 26	-299 ± 9
	Hydrophobic	43	0	-0.4 ± 0.6	-189 ± 7

Table S1. Statistics done during the last 10 ns of MD simulation of CT and 4Rep. Electrostatic (EELEC), and van der Waals (EVDW) energies expressed in kcal/mol.
